# A Narrative Review of Syncope Decision Rules: A Case for Point-of-Care Cardiac Ultrasound for Cardiac Syncope

**DOI:** 10.3390/jcm15103780

**Published:** 2026-05-14

**Authors:** Elaine Situ-LaCasse, Neil Wallace, David Wasiak, Pratham Patel, Srikar Adhikari

**Affiliations:** 1 Department of Emergency Medicine, University of Arizona College of Medicine, Tucson, AZ 85724, USA; nwallace@aemrc.arizona.edu (N.W.); dwasiak@aemrc.arizona.edu (D.W.); sadhikari@aemrc.arizona.edu (S.A.); 2 University of Arizona College of Science, Tucson, AZ 85721, USA; prathampatel05@gmail.com

**Keywords:** syncope, point-of-care ultrasound, cardiac ultrasound, pulmonary embolism

## Abstract

Syncope is a common complaint among Emergency Department patients. There are innumerable causes for syncope, and it poses a diagnostic challenge for Emergency Medicine (EM) physicians. Currently, several clinical decision rules (CDRs) for risk stratification of patients presenting with syncope depend on the circumstances surrounding the syncopal event, past medical history, vital signs, and results of basic diagnostic studies. However, the basis of these clinical decision tools is unreliable, such as the past medical history or nonspecific findings for various disease processes that can cause syncope. Given risk factors from unreliable sources, this raises the question: are we misclassifying our syncope patients? We posit in this narrative review that there is a role for a bedside diagnostic test, performed by the Emergency Medicine clinician, to assist with accurate risk stratification of syncope-causing pathologies: point-of-care cardiac ultrasound (POCCUS).

## 1. Background

Approximately 1–3% of all Emergency Department visits are for syncope or near-syncope [1]. Syncope is defined as a transient loss of consciousness caused by cerebral hypoperfusion. The differential diagnosis for syncope is vast, from benign to life-threatening causes, and this poses a dilemma for EM clinicians. Because of this, syncope patients can undergo extensive evaluations, including inpatient stays. The cost of caring for syncope patients is high and growing, resulting in more than $2.4 billion in hospital costs per year [2].

The current risk stratification tools factor in prodrome, age, electrocardiogram (EKG) findings, past medical history, laboratory studies, and vital signs. The Canadian Cardiovascular Society Position Statement in 2020 stated that syncope decision rules are not widely adopted because of important methodological limitations [3], and these decision rules did not show better diagnostic or prognostic yield in outcome prediction when compared to clinician’s medical decision making alone [4]. None of these clinical decision rules (CDRs) utilize imaging studies, and the imaging modality most well-suited for evaluating the cardiac status of syncope patients in the Emergency Department is the point-of-care cardiac ultrasound (POCCUS). There is currently a paucity of research in this area, and no established protocols to integrate POCCUS into evaluating patients with syncope to rule-in or rule-out a cardiac cause (arrhythmic, structural, cardiopulmonary).

POCCUS is one of the most commonly performed point-of-care ultrasound applications, used to evaluate chest pain, sepsis, hypotension, etc. [5]. The assessment for hypotension, which includes inferior vena cava (IVC), gross left ventricular (LV) function, and pericardium, takes an average of 6 min to perform [6]. Kapoor 1983 et al. showed that the 12-month mortality rate in syncope patients is 14%, with the highest percentage (30%) in patients with a cardiovascular cause [7]. The European Society of Cardiology 2018 Guidelines for the diagnosis and management of syncope recommends an echocardiogram for previous known heart disease or data suggestive of structural heart disease or syncope secondary to cardiovascular cause, which is done by a dedicated echocardiographer [8], but there was no mention of cardiac ultrasounds performed by physicians at the bedside. Using point-of-care ultrasound at the bedside gives physicians the opportunity to improve and expedite the risk stratification of syncope patients who present with limited to no known medical history.

POCCUS is not currently incorporated into syncope evaluation frameworks due to: (1) the wide range prevalence (6–30%) of structural causes detectable by ultrasound [9]; (2) the absence of standardized protocols and high-quality outcome data; and (3) variability in availability and operator dependence [10]. However, increasing accessibility and expanding evidence in emergency settings suggest a potential evolving role as a targeted adjunct to current risk stratification tools to improve early identification of high-risk structural causes of syncope. Our narrative review will provide an overview of the current CDRs for syncope evaluation and recommend the integration of POCCUS into risk stratification tools.

## 2. Literature Review Approach

This narrative review was conducted using searches of PubMed for articles related to syncope, clinical decision rules, point-of-care ultrasound, and Emergency Department evaluation. Relevant studies were identified using combinations of keywords including “clinical decision rules,” “syncope,” “point-of-care ultrasound,” “cardiac ultrasound,” and “risk stratification.” Articles were selected based on relevance to the topic, with an emphasis on studies published in the last 20–25 years, as well as key guideline documents.

## 3. Current Risk Stratification Tools

Current risk stratification rules have significant limitations despite their widespread use in syncope risk stratification. Multiple CDRs rely heavily on patient-provided history and past medical history, as evidenced by Table 1. This approach is inherently flawed because patients cannot or do not provide accurate information surrounding the syncopal event. According to the 2018 census, approximately 27.5 million people lacked health insurance, increasing the risk of undiagnosed conditions due to limited access to care [11]. Therefore, patients may be presenting to the ED with undiagnosed cardiac conditions that would not be detected from CDRs alone.

Moreover, all the CDRs include history of heart disease or heart failure as risk factors, and inclusion of these risk factors are dichotomous. However, this does not factor in progression or worsening or improvement of chronic conditions. Each of the CDRs also include abnormal EKGs or EKG changes as a risk factor for high-risk syncope, but the criteria are not consistent in the definition of “concerning” EKG changes. Additionally, changes in EKG can be unclear in their chronicity and may not contribute to the patient’s presenting symptoms, making EKG changes nonspecific. In Albassam et al.’s Rational Clinical Examination Systematic Review, of the 4317 patients included in their analysis, the finding of atrial fibrillation or flutter had a sensitivity of 0.13 (0.06–0.20) and a specificity of 0.98 (0.96–1.0) in detecting cardiac syncope [12], which showed no single clinical or EKG finding can diagnose a cause for syncope. Risk stratification relies on the integration of multiple factors.

The San Francisco Syncope Rule is a widely cited tool to predict mortality in patients who present to the ED with syncope. Quinn et al. prospectively validated the San Francisco Syncope Rule (SFSR), and in their study of 791 consecutive syncope ED visits, the clinical decision rule was 98% sensitive and 56% specific for predicting adverse events [13]. Their results showed 6.8% had a serious outcome after the ED visit, with arrhythmia and myocardial infarction at the top of the list. Cardiogenic syncope increases the risk of death by two-fold and also increases the risk of cardiovascular events [14]. However, when the SFSR was applied to the elderly population of the ED (> age 65), the sensitivity decreased to 76.5%, and specificity also dropped to 36.8% [15]. The elderly population is one of the most vulnerable populations we care for in the ED, and the ability to risk stratify this group of patients is critical, especially since other scores, such as the OESIL risk score, show that advanced age (>65 years) is one of the most predictive factors for death during the follow-up period [16].

The OESIL (Osservatorio Epidemiologico sulla Sincope nel Lazio) risk score was prospectively validated by Colivicchi et al. with the primary endpoint as death from any cause within 12 months of study participation [16]. Univariate predictors of death were age >65, syncope without prodromes, cardiovascular disease in clinical history, and abnormal EKG, sharing similar limitations of additional syncope rules. Syncope without prodrome has been considered as cardiac syncope, which yields a negative prognosis [16].

The EGSYS (Evaluation Guidelines in Syncope Study) Score created by Brignole et al. 2006 based on the European Society of Cardiology (ESC) syncope management guidelines in 2006 utilized an interactive online decision-making system to assist physicians with the management of a patient who presents with syncope [17]. Based on this, Del Rosso et al. prospectively validated the study in a multicenter study with 516 patients by determining the probability of cardiac syncope and mortality [18]. Out of the 516 patients, the majority (67%) had a neurally mediated cause for syncope, and 79 (15%) patients had cardiac syncope, with 29% of those patients having a mechanical cause: acute coronary syndrome, aortic stenosis, pulmonary embolism, pericardial tamponade, and Eisenmenger syndrome. Several life-threatening structural causes of syncope—including tamponade, severe ventricular dysfunction, and right ventricular strain—are detectable using POCCUS, suggesting that integration of this modality may facilitate earlier bedside recognition [19].

The Canadian Syncope Risk Score was more recently validated in a prospective, multicenter Emergency Department study by Thiruganasambandamoorthy et al. to guide management of syncope patients whose serious causes were not identified during their initial ED visit [20]. In this study, 160 (4%) patients out of 3979 patients were found to have serious cardiac outcomes (arrhythmias, cardiac and non-cardiac serious outcomes, such as ACS, PE, etc.) identified during their initial ED evaluation. After their initial ED evaluation and disposition, 139 patients (3.6%) had serious outcomes.

Elderly patients who present with syncope have an increased risk of serious outcomes, and the FAINT Score was created to evaluate older adults who present with syncope to the Emergency Department [21]. In this prospective observational study of 3177 older patients (60 or older), the FAINT Score had a sensitivity of 96.7% and specificity of 22.2% for predicting all-cause mortality or serious cardiac outcome (significant cardiac arrhythmia, myocardial infarction, new diagnosis of structural disease, or cardiac intervention) at 30 days. Interestingly, the FAINT score study did not include patients with pulmonary embolism, focusing only on cardiac outcomes. However, pulmonary embolism deserves to be discussed specifically, since syncope is the presenting symptom in up to 20% of patients with pulmonary embolism (PE) [22].

Life-threatening causes of syncope are usually of cardiac origin, which includes acute PE [23], and PE has been identified in 2.2–35% of syncope patients [24]. The proposed mechanism for syncope secondary to PE is unclear, but the primary theory is that a large clot burden causes right ventricular (RV) dysfunction, leading to decreased cardiac output, hypotension, and decreased cerebral blood flow [25]. In patients with acute PE and syncope, there is a strong association between syncope and cardiogenic shock and low systolic blood pressure (<90 mmHg) [24]. Because pulmonary embolism is a common disease, seen in 1 in 1000 patients [26], and 1 in 6 patients with PE present with syncope, it is important to identify this patient subset [27]. In a recent meta-analysis, de Winter et al. concluded that there is an association between short-term mortality and syncope in patients with PE [22]. There is a 4% increase in short-term mortality when patients present with syncope in the setting of PE.

The current clinical decision rules demonstrate several shortcomings. They are limited in evaluation of mechanical causes of syncope, do not factor in explicit risks or findings for pulmonary embolism, utilize historical features that have questionable accuracy, dependent on patient-provided information, and do not account for progression of underlying diseases and exacerbations. This is an area where bedside cardiac ultrasound can provide both sensitive and specific information to direct management and augment current CDRs.

**Table 1:** Clinical decision rules.

Clinical Decision Rule	Risk Factors	Scoring	End Point
San Francisco Syncope Rule [13]	- History of heart failure - Hematocrit < 30% - Abnormal EKG - Shortness of breath - Systolic blood pressure < 90 mmHg on presentation	Low risk: 0 risk factors High risk: ≥1 risk factor	7-day serious adverse events
OESIL Score 1999 [16]	- Age > 65 years - History of cardiovascular disease - Abnormal EKG - Syncope without prodrome	0–4 (1 point for each risk factor)	12-month all-cause mortality
EGSYS Score [18]	- Abnormal EKG and/or heart disease (+3) - Palpitations before syncope (+4) - Syncope during effort (+3) - Syncope in supine position (+2) - Autonomic prodromes (−1) - Predisposing and/or precipitating factors (−1)	Sum of (−) and (+) points assigned to each risk factor. Low risk of cardiac cause and mortality: <3 High risk: ≥3	21–24-month mortality and cardiac syncope probability
Canadian Syncope Risk Score [20]	- Predisposition to vasovagal symptoms - Heart disease history - Systolic blood pressure < 90 or >180 mmHg - Elevated troponin - Abnormal QRS axis - QTc > 480 ms - ED diagnosis	Sum of (−) and (+) points assigned to each risk factorTotal score [−3 (very low risk) to 11 (very high risk)]	30-day serious adverse events
FAINT Score [21]	- History of heart failure (+1) - History of cardiac arrhythmia (+1) - Initial abnormal EKG (+1) - Elevated pro B-type natriuretic peptide (+2) - Elevated high-sensitivity troponin (+1)	0–6 (1 or 2 points assigned to each risk factor)Patients classified as low risk (0) or nonlow-risk (≥1)	30-day all-cause mortality or serious cardiac outcome

## 4. Point-of-Care Cardiac Ultrasound

The prognosis of syncope is largely determined by the presence of underlying heart disease rather than the syncopal event itself. Population-based data from the Framingham Heart Study demonstrated that cardiac syncope is associated with significantly higher overall and cardiovascular mortality compared with vasovagal syncope. One-year mortality rates in patients with cardiac syncope range from 18 to 33%, whereas mortality in reflex syncope is generally less than 5% [28]. Accordingly, current ACC/AHA/HRS and ESC guidelines emphasize early identification of high-risk cardiac features to enable timely diagnostic evaluation and therapeutic intervention, such as pacemaker implantation, implantable cardioverter-defibrillator placement, or corrective structural procedures, all of which may substantially reduce morbidity and mortality [8,29]. Timely evaluation and intervention can be achieved by performing a POCCUS, such as left ventricular dysfunction, right heart strain, pericardial effusion, IVC collapsibility, and severe valvular abnormalities.

None of the widely used risk stratification rules incorporate POCCUS in the clinical decision-making algorithm. Cardiac imaging in syncope evaluation is frequently performed by cardiology consultants after patients have been admitted to the hospital. In 2017, the American College of Cardiology, American Heart Association, and the Heart Rhythm Society stated that transthoracic echocardiogram (TTE) would be beneficial in patients who have an abnormal EKG or if there is a concern for structural cardiac problems or cardiac disease history [29]. Subsequent evaluations of syncope patients frequently include a cardiology-performed TTE.

Baugh et al. examined the variation in testing among elderly patients presenting to the Emergency Department [30]. They noted that 35% of syncope patients received an echocardiogram, and among the top five most commonly performed tests, the echocardiogram had the highest proportion of abnormal results at 22.1%. However, the echocardiogram accounted for the highest total costs at $672,648, and the cost per abnormal test is $3129, with each echocardiogram costing $514.65, but that does not factor in the associated hospitalization costs. For a limited cardiac ultrasound (CPT 93306) performed in the ED, the cost per ultrasound is lower at $481.53, according to the 2020 CMS Medicare Reimbursement rates [31].

Prior studies have demonstrated emergency physicians’ ability to perform limited cardiac ultrasound to evaluate LV contractility and ejection fraction [32]. In 2010, in conjunction with the American Society of Echocardiography, the American College of Emergency Physicians advocated for the use of POCCUS for the evaluation of pericardial effusion, relative chamber size, and global cardiac function [33]. Given that bedside cardiac ultrasounds are one of the most commonly performed point-of-care examinations, adding this diagnostic test to the evaluation of patients with syncope is not burdensome, especially when it can reveal vital information. Acquiring a patient’s history can be difficult under various emergent circumstances, and in the elderly population, it can be even more challenging. Lindner et al. studied taking medical history of elderly patients (>75 yo) in the ED, and 25% of them could not provide a complete basic medical history (medications, past medical history, allergies, and primary care physician) [34]. Past medical history can be limited, especially if the patient has not seen a physician or has limited access to care. Congestive heart failure may be first diagnosed after presenting to the ED with a syncopal episode.

Early identification of pericardial effusion using point-of-care cardiac ultrasound (POCCUS) is also crucial, particularly in hemodynamically unstable patients, as it allows rapid bedside detection of potentially life-threatening cardiac tamponade. POCCUS enables immediate visualization of pericardial fluid and assessment of hemodynamic compromise through findings such as right atrial or right ventricular diastolic collapse and inferior vena cava plethora, which are key echocardiographic features of tamponade physiology [35,36]. In emergency and critical care settings, focused cardiac ultrasound has been shown to expedite diagnosis, guide urgent pericardiocentesis, and improve time-sensitive clinical decision-making [37,38]. Early bedside diagnosis is therefore essential to reduce delays in intervention and potentially decrease morbidity and mortality associated with untreated cardiac tamponade [35]. The development of pericardial effusion is insidious, as the patient may present with fatigue, shortness of breath, hypotension, and possibly syncope. The decision to intervene with a procedure urgently or emergently can be determined by performing POCCUS.

In the cases of suspected PE causing abnormal vital signs (tachycardia of ≥100 bpm or hypotension < 90 mmHg), focused cardiac ultrasound was 92% sensitive for PE [39]. With abnormal vital signs, findings of right ventricular dysfunction (RVD) are more likely seen on POCCUS. The typical findings for RVD and elevated right-sided heart pressures are dilation of the RV, D-sign/septal flattening or bowing, McConnell’s sign, decreased tricuspid annular plane systolic excursion (TAPSE), and with worsening RVD, tricuspid regurgitation. With PE identified in 2.2–35% of syncope patients [24], performing POCCUS to evaluate for signs of RVD is well worth the time.

## 5. Future of POCCUS in Syncope Risk Stratification

Syncope risk stratification is a complex and challenging task, as evidenced by the numerous clinical decision rules and studies that have sought to validate them. In the Emergency Department, where physicians are tasked with making decisions based on limited or unreliable information, an accessible, quick, highly informative test for risk stratification can be immensely helpful. It serves as an objective, rapid bedside tool for evaluating structural and functional cardiac abnormalities that may underlie syncope. Incorporating POCCUS into clinical practice, such as left ventricular dysfunction, right heart strain, or pericardial effusion, into current clinical decision rules (CDRs) could enhance risk stratification by providing immediate physiologic data. Because of the limited literature on how well Emergency Medicine providers classify syncope with the addition of POCCUS, it is unclear how POCCUS will change risk stratification in this patient population or whether we may have been misclassifying patients with current tools. However, it can be incorporated as an additional data point into the existing syncope rules to better stratify our patients rather than a standalone tool. More research must be done in this area to improve patient outcomes and resource utilization.

**Figure 1:**
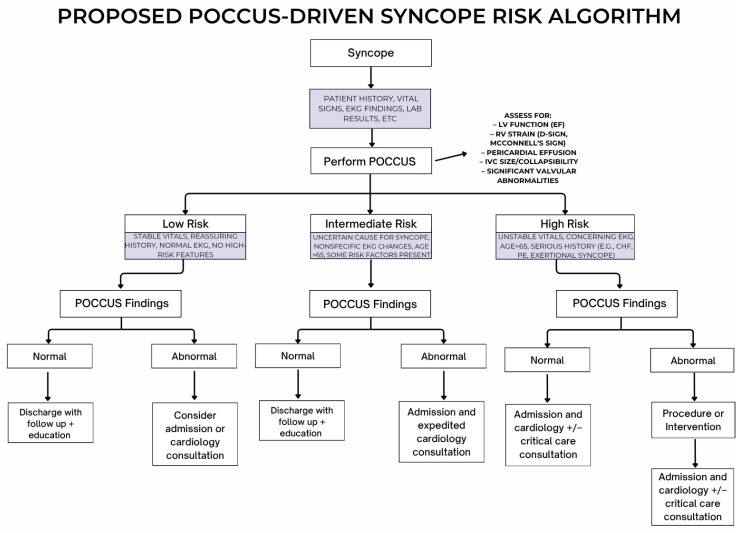
Proposed point-of-care cardiac ultrasound syncope risk algorithm, which has not yet been validated.

Figure 1 shows a proposed (not yet validated) algorithm incorporating POCCUS into the usual evaluation of a patient who presents with syncope and the subsequent patient disposition. After the patient with syncope presents to the ED, they will undergo a full patient history and physical examination along with EKG, blood work, etc. Then the patient will undergo a POCCUS exam. A POCCUS exam in syncope should assess for: (1) pericardial effusion/tamponade, (2) gross left ventricular dysfunction, (3) right ventricular strain (D-sign, McConnell’s sign), IVC (size, collapsibility), and (4) significant valvular abnormalities. The absence of abnormal POCCUS findings combined with stable vitals, reassuring history, normal EKG, and lack of high-risk features (overall low risk) would mean the patient can be discharged with follow-up, education, and strict return precautions. If the POCCUS is abnormal, then admission or cardiology consultation should be considered.

If the patient is deemed to be an intermediate risk based on history, nonspecific EKG changes, age, and risk factors and has a normal POCCUS, then the patient could be discharged with close follow-up. If there are abnormal POCCUS findings, then admission and cardiology consultation are recommended. A normal POCCUS examination cannot independently exclude serious causes of syncope. Although point-of-care ultrasound has demonstrated utility in identifying structural and hemodynamic abnormalities in acute care settings, there is limited evidence supporting its impact on risk stratification or clinical outcomes in syncope, and its integration into standardized decision-making frameworks remains unvalidated. As such, the proposed algorithm should be considered conceptual and hypothesis-generating.

Per our protocol, if the patient is deemed high risk, the patient will need to be admitted regardless of what the POCCUS reveals, but the POCCUS may reveal a finding that would need immediate intervention, such as pericardial tamponade or severe right heart strain in PE. It can also refine differential diagnoses and guide early management in the ED or after admission. POCCUS alone does not rule out serious syncope conditions [40], but it is a useful tool to increase diagnostic accuracy and augment clinical assessments.

This suggested algorithm does not comment on whether cross-sectional imaging, such as computed tomography, is needed in the patient cases. This level of medical decision-making will be deferred to the treating physician.

Although we are promoting bedside cardiac ultrasound, there are also challenges to its widespread implementation. The barriers can be qualified personnel, equipment availability, and appropriate documentation. For emergency physicians who are not trained in POCCUS or have limited experience with point-of-care ultrasound, adding this step will be challenging, as they will need to seek additional training, which can be inconsistent across institutions. The American College of Emergency Physicians recommends 25–50 quality-reviewed POCCUS to achieve proficiency [41], which is challenging when there is no local expert to a provide quality-improvement review.

Point-of-care ultrasound is inherently operator-dependent, with diagnostic accuracy influenced by the clinician’s ability to acquire and interpret images, as well as integrate findings into clinical decision-making. Variability in training and experience contributes to differences in performance, even among emergency physicians [10].

As mentioned previously, POCCUS findings should be interpreted within the broader clinical context of risk stratification rather than in isolation, as findings such as right ventricular dilation or reduced ejection fraction may lack specificity [42]. Concerns regarding potential misdiagnosis, overuse, and inappropriate reliance on POCUS have also been raised in the literature [42]. Overreliance on POCUS without appropriate clinical context may lead to diagnostic error or inappropriate management [42].

Ultrasound-guided central venous catheter placement is considered the standard of care, so EDs should have an accessible ultrasound machine; however, the appropriate transducer may not be available, as the phased-array transducer is preferred for cardiac evaluation. Acquisition of an ultrasound machine or additional transducers may be cost-prohibitive, as phased-array transducers may cost over $4500 new.

Additionally, for documentation and medicolegal purposes, images of the POCCUS examinations performed should be saved and retrievable for medical record review and/or quality improvement (QI). This would require dedicated time and personnel to maintain such a repository and perform QI, both of which require expertise and financial expenditure.

## 6. Conclusions

Syncope remains a complex and high-stakes presentation in the Emergency Department, and current risk stratification tools are limited by relying on subjective history and nonspecific findings. These limitations raise concern for misclassification, particularly in patients with undiagnosed or underlying cardiac pathology.

POCCUS provides a rapid, objective bedside assessment that can identify critical conditions, including pericardial tamponade, right ventricular dysfunction, and cardiomyopathy. By providing real-time data, POCCUS can enhance diagnostic accuracy, assist medical decision-making, and improve patient disposition.

Although implementation barriers exist, they are becoming less problematic as ultrasound training and accessibility expand. POCCUS should not be viewed as a universal screening tool for syncope, but rather as a targeted adjunct in patients where structural or hemodynamic causes are suspected. Incorporating POCCUS as an adjunct to existing clinical decision rules represents a practical step toward more accurate, data-based syncope evaluation. Adoption of POCCUS in syncope assessment should be prioritized to define its role within standardized risk stratification frameworks, as it offers a meaningful opportunity to improve risk stratification, patient outcomes, and resource utilization in the Emergency Department.

## Data Availability

Not applicable.

## Abbreviations

The following abbreviations are used in this manuscript:

EM	Emergency Medicine
ED	Emergency Department
CDR	Clinical decision rules
POCCUS	Point-of-care cardiac ultrasound
IVC	Inferior vena cava
LV	Left ventricle
SFSR	San Francisco Syncope Rule
OESIL	Osservatorio Epidemiologico sulla Sincope nel Lazio
EGSYS	Evaluation Guidelines in Syncope Study
ESC	European Society of Cardiology
RV	Right ventricle
RVD	Right ventricular dysfunction
PE	Pulmonary embolism
ACC	American College of Cardiology
AHA	American Heart Association
HRS	Heart Rhythm Society
TTE	Transthoracic echocardiogram
CPT	Current procedural terminology
CMS	Centers for Medicare and Medicaid Services
EKG	Electrocardiogram
Bpm	Beats per minute
mmHg	Millimeters of mercury
TAPSE	Tricuspid annular plane systolic excursion
QI	Quality improvement
